# The Evolving Role of ^18^F-FDG PET/CT in Diagnosis and Prognosis Prediction in Progressive Prostate Cancer

**DOI:** 10.3389/fonc.2021.683793

**Published:** 2021-07-29

**Authors:** Kai Shen, Bo Liu, Xiang Zhou, Yiyi Ji, Lei Chen, Qi Wang, Wei Xue

**Affiliations:** ^1^State Key Laboratory of Oncogenes and Related Genes, Department of Urology, Renji Hospital, School of Medicine, Shanghai Jiao Tong University, Shanghai, China; ^2^Department of Nuclear Medicine, Renji Hospital, School of Medicine, Shanghai Jiao Tong University, Shanghai, China

**Keywords:** ^18^F-FDG, PET/CT, ^68^Ga-PSMA, glucose metabolism, prostate cancer

## Abstract

Positron emission tomography/computed tomography (PET/CT) is widely used in prostate cancer to evaluate the localized tumor burden and detect symptomatic metastatic lesions early. ^18^F-FDG is the most used tracer for oncologic imaging, but it has limitations in detecting early-stage prostate cancer. ^68^Ga-PSMA is a new tracer that has high specificity and sensibility in detecting local and metastatic tumors. But with the progression of prostate cancer, the enhancement of glucose metabolism in progressive prostate cancer provides a chance for ^18^F-FDG. This review focuses on PET/CT in the detection and prognosis of prostate cancer, summarizing the literature on ^18^F-FDG and ^68^Ga-PSMA in prostate cancer and highlighting that ^18^F-FDG has advantages in detecting local recurrence, visceral and lymph node metastases compared to ^68^Ga-PSMA in partial progressive prostate cancer and castration-resistant prostate cancer patients. We emphasize ^18^F-FDG PET/CT can compensate for the weakness of ^68^Ga-PSMA PET/CT in progressive prostate cancer.

## Introduction

The incidence and mortality of prostate cancer rank first among all cancers, accounting for 1/10 of all cancer deaths in the United States (1). After receiving effective treatment such as surgery and radiotherapy, 2/5 of patients with prostate cancer experience a detectable rise in the serum prostate-specific antigen (PSA) level in the next 10 years from the first treatment, which includes local recurrence and metastatic disease (2, 3). With the increased management of hormone therapy, nearly all patients eventually develop castration-resistant prostate cancer (CRPC), which is the main cause of disease-related death (4, 5). Some CRPC patients would progress into a rare pathological subtype called neuroendocrine prostate cancer (NEPC). The resistance to treatment is caused by following the mechanisms as TP53 mutation, AR amplification, or mutations or RB1 loss. For the difficulty in early diagnosis and prediction of prognosis, PSA monitoring along with radiologic evaluations is standard for tumor burden assessment. Multiple imaging techniques are used for diagnosis or staging, assessment of treatment, and prognosis prediction including Magnetic Resonance Imaging (MRI), Computed Tomography (CT), ultrasound, Single-Photon Emission Computed Tomography (SPECT), and PET/CT. Guidelines recommend MRI or CT for staging, detecting lymph node metastases, and local recurrence. Besides traditional anatomic imaging, nuclear imaging-based PET/CT is a rapidly developing field for compensating the limitation of whole-body evaluation and precise prognosis prediction. The most commonly used tracer is ^18^F-fluorodeoxyglucose (FDG), and the newer tracer ^68^Ga-prostate-specific membrane antigen (PSMA) also provides good detection. ^18^F-FDG PET/CT is shown to be a useful prognostic tool in selected patients with advanced disease (6–8). Intraprostatic uptake of ^18^F-FDG imaged by PET/CT suggests that aggressive behavior and castration resistance with increased glucose uptake (9, 10). In addition, ^18^F-FDG PET/CT has played a significant role in many kinds of cancer for a long time. ^18^F-FDG PET/CT is widely used in lymphoma, breast cancer, lung cancer, esophageal cancer, colorectal cancer, and many other cancers (11); it performs well in diagnosis, staging, prediction of recurrence, and prognosis prediction of the above diseases.

This article mainly summarizes the former research of the mechanism and usage of ^18^F-FDG and ^68^Ga-PSMA PET/CT in prostate cancer. We compare the pros and cons of ^18^F-FDG and ^68^Ga-PSMA in localized prostate cancer. We then focus on exploring the potential of ^18^F-FDG PET/CT in aggressive and progressive prostate cancer. In addition, in order to maintain a comprehensive review, we also briefly summarized the clinical use of choline PET in prostate cancer.

## The Basic Mechanism of Detecting Prostate Cancer

PET/CT is a non-invasive examination for diagnosis, and many radiotracers are widely used in different malignant tumors. PET-based radiotracers, ^18^F-FDG, is the most commonly used radiotracer for oncologic imaging and is based on the increased glucose metabolism in malignant tumors (12, 13). FDG, a glucose analog, is transported into the cell through GLUTs and then phosphorylated by hexokinases to FDG-6-phosphate which then is stored within the cells. Malignant cancers convert to an increased rate of glycolysis at an advanced stage named Warburg effects in the transformation process (14, 15). Androgens enhance glucose metabolism by modulating glycolytic-related genes (16). Furthermore, Glucose uptake and metabolism rely on the transporter GLUT family and hexokinase which are involved in tumor progression and overall survival (17–20). Compared with normal epithelial cells, cancer cells enhancee glycolysis, showing higher SUVmax in PET/CT. The expression of GLUT1 correlated with disease progression, and tumor cells enhance glycolysis with the elevation of GLUT1 (19). A significant association was found between GLUT1 expression levels and SUVmax level (p = 0.005), lymph node status (p = 0.05), volume of cancer (p = 0.01), CRPC disease progression (p = 0.02), and metastases development (p = 0.04). Prostate cancer upgrades the expression of hexokinase2 when progressing to CRPC. Several studies have proven that Pten/p53 deficient mice elevated levels of hexokinase2 and its binding to mitochondrial enhanced enzyme activities (21, 22). ^18^F-FDG is used as an auxiliary method to analyze glucose metabolism in prostate cancer cells and find new targets and methods for the diagnosis and treatment of prostate cancer.

The new tracer PSMA in prostate cancer is a transmembrane protein with a 707-amino-acid extracellular portion located in the apical prostate cell surrounding ducts. In the physiological statue, PSMA shows accumulation in the salivary gland, the liver, the spleen, the small bowel, and the urinary tract. In normal prostate, PSMA localizes in the cytoplasm and apical side of the epithelium surrounding prostatic ducts. Dysplastic or neoplastic transformation transfers PSMA from the apical membrane to the luminal surface of the ducts, and this has been detected by PET/CT (23). PSMA has become the standard method for diagnosing and staging prostate cancer. Several meta-analyses concluded ^68^Ga-PSMA PET/CT improved detection of localized prostate cancer and metastases (24, 25).

## Localized Prostate Cancer

**Figure 1:**
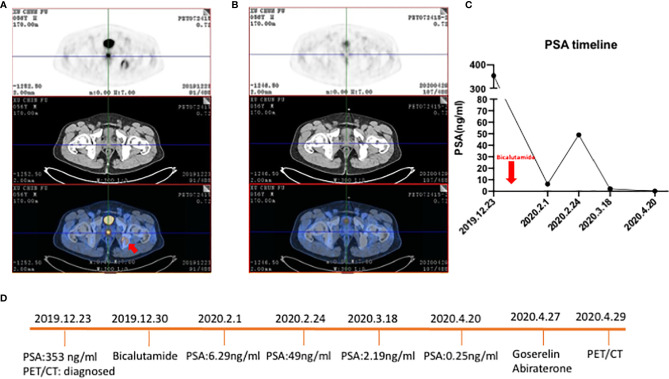
^18^F-FDG PET/CT of a 56-year-old patient with primary prostate cancer who was sensitive to ADT. **(A)** Patient was diagnosed with primary prostate cancer and an oligometastatic lesion in the right ischium (red arrow) with an osteogenic bone lesion. SUVmax of primary prostate cancer was 7.2. **(B)** After ADT tumor burden was significantly decreased and the oligometastatic lesion regressed. **(C)** Line graph of PSA level. The red arrow was the timepoint patient taking bicalutamide. **(D)** Timeline of patient underwent ADT and time for PET/CT.

^18^F-FDG PET/CT acts as a detection tool to detect metastases during the observation period but has low sensitivity in localized prostate cancer. At present, many viewpoints believed that for localized prostate cancer, compared with observation, active radical prostatectomy or external-beam radiotherapy didn’t have much effect on the mortality of patients but it could control the progression of some prostate cancer (26, 27). Although the current data showed that the detection of ^18^F-FDG PET/CT had some limitations, there were still some studies suggesting the value of ^18^F-FDG PET/CT. Serendipitous high focal ^18^F-FDG uptake in the prostate gland in several case reports suggested that this imaging tool was useful for specific types of tumors (5, 28–30). In an investigation among 47,109 men who underwent ^18^F-FDG PET in a 10-year period, 1,335 (2.83%) showed incidental prostatic ^18^F-FDG uptake, and 99 of these men underwent prostate biopsy (31). Prostate cancer occurred in 1 of 26 patients (3.8%) with serum PSA<2.5 ng/mL, compared with 40 of 67 patients (59.7%) with serum PSA≥2.5 ng/mL. It revealed that patients with high ^18^F-FDG uptake in the prostate should be further evaluated by the measurement of serum PSA and prostate biopsy. Despite the low sensitivity, ^18^F-FDG could predict therapy effects for patients who exhibiting high absorption of ^18^F-FDG in tumor lesions. We looked at a patient who was primarily diagnosed with prostate cancer with oligometastatic lesions, and he was sensitive to androgen deprivation therapy (**Figure 1**). With the continuous decrease of PSA level, the FDG accumulation decreased in the prostate and right ischium. New tracers, ^68^Ga-PSMA and ^11^C-choline, have higher sensitivities in localized prostate cancer. In primary prostate cancer, the sensitivity of PSMA was 40–95%, which correlated with the levels of serum PSA. When PSA was over 2ng/mL, the sensitivity elevated to 95% (24). A meta-analysis including nine studies and a total of 547 patients with primary prostate cancer found the sensitivity of PSMA ranging from 67–97% (32). This study pointed out that PSMA had higher sensitivity and specificity in detecting primary prostate cancer compared with conventional imaging examinations. Similarly, the sensitivities of ^11^C- and ^18^F-choline in the diagnosis of primary prostate cancer were 66–86.5%, and the specificities were 43–81%. However, some studies showed that choline PET could not distinguish between benign and malignant tumors, or between inflammatory and malignant tissue in microcarcinomas and small tumors (33–41). Besides, choline PET/CT has a limited role in staging and is only beneficial in the detection of distant metastases such as bone metastases (33).

There is the consensus that CT/MRI is preferred for detecting primary prostate cancer. PET/CT is not particularly satisfactory because of the lower or similar sensitivity and being less convenient and commercial in clinical practice. ^18^F-FDG, ^68^Ga-PSMA, and ^11^C-choline have relatively low sensitivity when PSA is lower than 2ng/mL and increase the sensitivity when PSA is over 2ng/mL. Patients whose PSA is over 2ng/mL are often required for biopsy to diagnose. For primary prostate cancer, PET/CT may act as an auxiliary method to evaluate the tumor burden and metastases.

## Progressive and Aggressive Prostate Cancer

After initial treatment as radical prostatectomy or androgen deprivation therapy (ADT), the disease progresses into metastasis, recurrence, or resistance to treatment. ^18^F-FDG PET/CT has been used as a commonly used means to observe the effects of treatment and surveillance together with CT, MRI, PSMA, and bone scan (42).

### Metastasis and Recurrence

PET/CT is a promising tool to identify lymph node metastases. Both ^18^F-FDG and ^68^Ga-PSMA PET/CT have high specificity in detecting metastases with direct therapy. Yi, C. et al. evaluated the efficacy of ^18^F-FDG in the detection of advanced prostate cancer and its metastases (43). In the detection of 26 patients with high-risk prostate cancer (Gleason score 8–10 or PSA> 20 ng/mL or clinical tumor extension ≥ T2c), the sensitivity and specificity for the diagnosis of lymph node metastases were 75% and 44.4%, respectively. This shows that ^18^F-FDG plays a role in asymptomatic people with high PSA and lymph node metastases. Moreover, ^18^F-FDG guides stage and restage of prostate cancer by detection of tumor burden and metastases. ^18^F-FDG has a high positive predictive value for untreated metastases in viscera but no lymph nodes (44). Beauregard et al. performed ^18^F-FDG PET/CT on 44 patients with known Gleason≥8 for the staging workup (9). High ^18^F-FDG uptake was found in the prostate gland, lymph nodes, and bone in 44%, 13%, and 6% of the patients, respectively. The absence or presence of intraprostatic ^18^F-FDG uptake was associated with a median cancer-free survival probability of 70.2% and 26.9% (P = 0.0097), respectively. ^18^F-FDG PET/CT can detect local and distant metastases with relatively high accuracy. Compared with ^18^F-FDG, ^11^C-choline PET/CT appears to have a better value for biochemical recurrence and restaging (45), but neither of them was satisfactory. Researchers focused on the potential utilization of ^68^Ga-PSMA to detect metastases and restage tumor burden accurately. Lars et al. assessed the diagnostic accuracy of ^68^Ga-PSMA before radical prostatectomy (46). Their work revealed the specificity of PSMA was 100%, but the sensitivity was only 33.3%. The author concluded the accuracy of PSMA based on the size of enlarged lymph. Another retrospective research analyzed the PSMA-positive metastasis with different nomograms (47). The sensitivity ranged from 22.3% to 40.5%. Some researchers compared ^68^Ga-PSMA with another new tracer. Ali. et al. compared ^68^Ga-PSMA to ^11^C-choline in 78 lesions from 32 patients and found ^68^Ga-PSMA could detect all lesions that were positive by ^11^C-choline; meanwhile, ^68^Ga-PSMA had a clearer tumor to background ratio (48). In summary, PET/CT is recommended for localizing metastases and identifying diagnoses to assist in surgery planning. Among these tracers, ^68^Ga-PSMA has proved high specificity in detecting lymph nodules lesions while ^11^C-choline has been recommended for bone metastasis in NCCN guidelines.

Recurrence means the failure of treatment and poor prognosis. ^18^F-FDG PET/CT can detect the recurrence of the disease earlier than traditional imaging methods, which may be more meaningful for the remedial treatment and prolonging the survival time of patients with prostate cancer. Jadvar, H. et al. reported a prospective investigation to assess the association of ^18^F-FDG PET/CT with time to hormonal treatment failure (THTF) in 76 men with metastatic castration-sensitive prostate cancer (8). The conclusion is that sum of SUVmax and the number of lesions derived from ^18^F-FDG PET/CT provided independent prognostic information on THTF in men with metastatic castration-sensitive prostate cancer. In addition, FDG accumulation in the prostate decreased in all patients 1–5 months after the initiation of hormone therapy (49). FDG had a 31% sensitivity in detecting biochemical recurrence after radical prostatectomy when the PSA level exceeded 1.9ng/mL and the sensitivity increased to 40% (50). These studies indicate that the sensitivity of detection for non-castrated resistant prostate cancer was relatively low. Judging from the listed data, many studies have pointed out the limited value of ^18^F-FDG in the detection of primary prostate cancer. For ^11^C-choline PET/CT, a study reported that the overall detection rate of biochemical recurrence was 45%. High PSA levels and advanced pathological stage were significantly associated with an increased risk of positive PET/CT findings (51). These features were similar to FDG PET imaging data, but the relationship with the Gleason score was not clear (52). ^68^Ga-PSMA has advantages in detecting recurrence compared to other tracers. A retrospective analysis included 294 patients and showed the sensitivity and specificity were both around 70%, and the SUVmax impacted therapeutic management (53). A meta-analysis showed PSMA-positive rate in patients was 68% and it was correlated with early biochemical recurrence (54). Several prospective studies report management changes after PSMA with biochemical recurrence of prostate cancer (55, 56). ^68^Ga-PSMA could clarify recurrence which improved management in patients with biochemical recurrence.

In summary, ^18^F-FDG and ^11^C-choline PET/CT have certain similarities in the detection of primary and recurrent tumors. Meanwhile, ^68^Ga-PSMA PET/CT performs better in the detection of lymph nodule metastases and recurrence than others. And it has the potential to assist therapeutic management in patients with biochemical recurrence.

### CRPC and NEPC

After ADT or castration, treatment response varies from months to years. The insensitivity to the castration treatment is inevitable and is termed CRPC defined as three consecutive rises in PSA with two rises of 50% above or progression of bone lesions or progression of soft tissue lesions (57). During the development of CRPC, the rise of serum PSA was termed as biochemical recurrence (BCR) and several researchers reported ^18^F-FDG PET/CT as a predictor of time to BCR.

High uptake of ^18^F-FDG in prostate cancer meant a shorter time to happen biochemical recurrence. Lavallee, E. et al. analyzed ^18^F-FDG PET/CT before prostatectomy in 148 prostate cancer patients with Gleason≥8 (10). In multivariate analysis, SUVmax≥4.6 in the prostate was associated with double the risk of biochemical recurrence 1 year after operation. The median biochemical recurrence-free survival (BFS) was 11.3 months while that of patients with lower SUVmax was 49.5 months. In addition, the high FDG uptake in the prostate was related to the shorter time of castration resistance after radical prostatectomy. The authors concluded that preoperative intraprostatic FDG uptake can predict BFS and castration resistance following radical prostatectomy in patients. It revealed that increased utilization of ^18^F-FDG identified invasive prostate cancers.

Castration resistance prostate cancer cells have higher ^18^F-FDG uptake and glucose metabolism than primary tumors, which means they are more malignant. Prognosis of patients with CRPC correlated inversely with SUVmax (58). Fox et al. reported FDG along with flurodihydrostosterone (FDHT) PET/CT to distinguish patients for sensitivity to androgen receptor signaling inhibitors (59). The results showed that FDHT positive or FDG positive had an independent negative effect. FDHT-negative with FDG positive was most insensitive to ADT (hazard ratio [HR], 1.11; 95% CI, 1.05-1.16; P < .001), followed by FDHT-positive with FDG positive (HR, 1.05; 95% CI, 1.03-1.06; P < .001).

CRPC would inevitably develop into metastatic castration-resistant prostate cancer (mCRPC). Both PSMA and FDG were good predictions on prognosis and treatment response in mCRPC. In a prospective investigation, parameters derived from baseline ^18^F-FDG PET/CT were associated with overall survival (OS) in Eighty-seven castrate-resistant metastatic prostate cancer patients (7). The authors observed that the fourth-quartile range of the sum of SUVmax was related to the shortest OS. In another study, the authors used the CAPRA-S prognostic tool to conclude that the absence and presence of FDG uptake in the prostate were associated with a median 5-year cancer-free survival rate of 70.2% and 26.9% (P=0.0097), respectively (9). The expression of PSMA portended a poor prognosis in patients with mCRPC. High radiographic PSMA uptake had a shorter OS than low PSMA uptake (15.8 to 22.7 months) (60). ^68^Ga-PSMA had a sensitive response to therapy in mCPRC patients. Compared to the serum PSA level, 52.6% of patients showed partial remission on PSMA while 23.7% of serum PSA. Median OS stratified to PSA/PSMA response was 25.6/25.6 months (61).

Several researchers focused on the rare pathologic type, neuroendocrine prostate cancer, as more aggressive prostate cancer with castration resistance and rise of NE markers. Bakht, M. K. et al. evaluated the association between neuroendocrine gene signature and ^18^F-FDG uptake-associated genes including GLUTs and hexokinases, with the goal of providing a genomic signature to explain the reported ^18^F-FDG avidity of PSMA-suppressed tumors (20). Their work demonstrated that a neuroendocrine gene signature is associated with differential expression of genes encoding GLUT and hexokinase proteins. The authors concluded that alteration of ^18^F-FDG uptake-associated genes correlated positively with higher glucose uptake in AR- and PSMA-suppressed tumors. This suggested that when the detection of prostate cancer by PSMA was not ideal and ^18^F-FDG PET/CT played a more important role. Spratt et al. reported high SUVmax in NEPC bone lesions and soft tissue lesions was associated with a shorter survival time (62). Stratified by the median survival from NEPC diagnosis, patients who survived below 2.2 years had more PET avid bone (8 *vs.* 2, P = 0.06) and soft tissue lesions (7 *vs.* 1, P = 0.01), higher average SUVmax of bone (5.49 *vs.* 3.40, P = 0.04), and soft tissue lesions (8.02 *vs.* 3.90, P = 0.0002).

## ^18^F-FDG *Versus*^68^Ga-PSMA

It is widely accepted that ^68^Ga-PSMA PET/CT has more considerable accuracy in diagnosis and staging in primary prostate cancer than ^18^F-FDG (63). In a study focusing on the sensitivity of ^68^Ga-PSMA in evaluating biochemical recurrence, the total positive predictive values of the prostate, pelvic lymph nodes, extra-pelvic lymph nodes, bones, and distant organs were 28%, 38%, 13%, 22%, and 5%, respectively (64). Multiple studies have revealed that PSMA needs to improve sensitivity but has high specificity for detection of nodal metastases in intermediate-to-high-risk prostate cancer.

**Figure 2:**
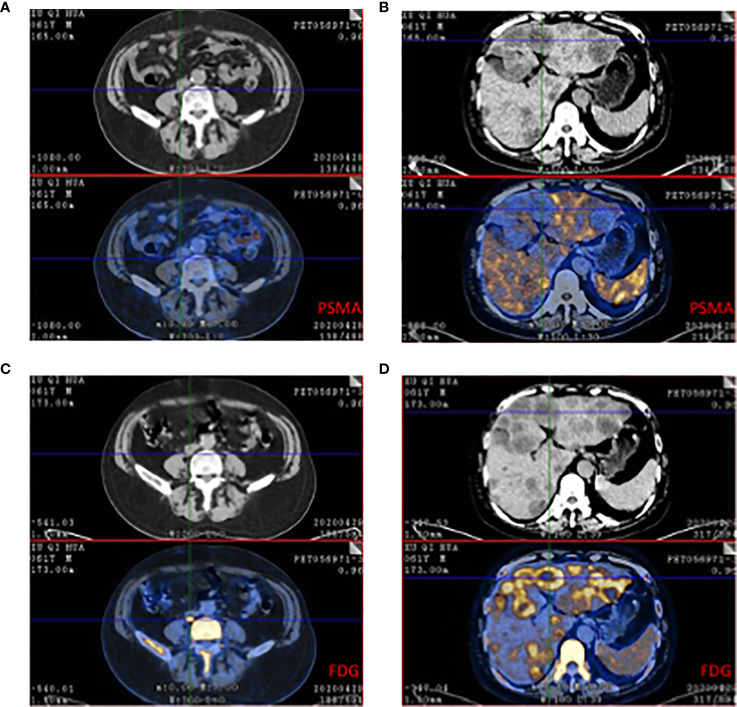
^68^Ga-PSMA and 18F-FDG PET/CT of a 61-year-old patient who was resistant to ADT and had widespread metastases in lymph node and liver. **(A)** CT showed the enlarged lymph node while PSMA expression was low. **(B)** Multiple low-intensity lesions were in the whole liver and the largest one was in the left hepatic lobe. The PSMA PET/CT showed low absorption in the suspicious lesion. **(C)** The same plane compared to A, FDG showed high absorption beside the vertebra. The size was 25*18mm and Sova=14.1. **(D)** The same plane compared to B, FDG showed heterogeneous enhanced in liver and SUVmax=4.8-11.0.

Plenty of studies have shown ^68^Ga-PSMA PET/CT had advantages in evaluating local recurrence or distance metastases but in CRPC PSMA had some limitations. A prospective single-arm clinical trial focused on accuracy in localizing recurrent prostate cancer. The data showed 75% of recurrent prostate cancer were positive and detection rates increased with PSA: 38% for <0.5 ng/mL, 57% for 0.5 to <1.0 ng/mL, 84% for 1.0 to <2.0 ng/mL, 86% for 2.0 to <5.0 ng/mL (n = 158), and 97% for ≥5.0 ng/mL (n = 173, P <.001) (65). This study demonstrated PSMA had high sensitivity in recurrent prostate cancer while it also showed PSMA might neglect tumor lesions for patients with low PSA. There are still some missed lesions because of the negative PSMA. A 61-year-old mCPRC patient had widespread metastases (**Figure 2**). ^68^Ga-PSMA showed negative in suspicious lesions in the liver while ^18^F-FDG exhibited high enhancement. This case demonstrated for some mCRPC patients FDG still had a role in risk stratification and recognizing metastatic lesions. A prospective trial included 37 patients which underwent both FDG and PSMA PET/CT (66). Of all 114 lesions, 81 were PSMA+FDG+, and 33 were PSMA-FDG+. PSMA-FDG+ lesions had a poor prognosis and resistance to castration. PSMA was a more sensitive and specific agent in prostate cancer, but in castration-resistant lesions, the sensitivity would reduce and mean more malignant lesions (67–69). Several case reports observed heterogeneity results that relied on pathological and clinical grading of prostate cancer. Some castration-resistant cancers showed PSMA negative and neuroendocrine tumors showed FDG and DOTATATE positive (68–70).

In the detection of primary and metastatic prostate cancer, the overall sensitivity and specificity of ^68^Ga-PSMA PET/CT are significantly higher than ^18^F-FDG PET/CT, but this does not mean that PSMA PET/CT has an excellent performance in all types of prostate cancer and all clinical environments, on the contrary, ^18^F-FDG plays a crucial role in CRPC and NEPC. For patients with negative ^68^Ga-PSMA, ^18^F-FDG has a diagnostic value correlated with PSA and the Gleason score (71). With the continuous improvement of the status of ^68^Ga-PSMA in the detection of prostate cancer, the auxiliary role of ^18^F-FDG should not be underestimated. The combination of ^68^Ga-PSMA and ^18^F-FDG may be more beneficial to the treatment and prognosis of patients.

## Conclusion

PET/CT has an important role in initial diagnosis, staging, and recurrence surveillance in a variety of cancers. The conventional tracer ^18^F-FDG was efficient for detecting lesions that maintained high glucose metabolism both in the primary tumor and metastases. Several clinical guidelines, like myeloma and lymphoma, recommended ^18^F-FDG PET/CT in diagnosis (72, 73). In solid tumors, ^18^F-FDG showed its high sensitivity for detecting metastases (74).

Prostate cancer is characterized by slow development and low glucose metabolism. In the 2020 prostate cancer NCCN guideline, CT/MRI is the priority when it comes to identifying primary lesions and evaluating the tumor volume. Conventional molecular imaging as ^18^F-FDG PET/CT should not be used routinely for staging in primary prostate cancer. In guidelines, new tracers such as ^11^C-choline can be used to detect small volume disease in soft tissues and bone. ^68^Ga-PSMA PET/CT is under several clinical trials and shows its advantages in both specificity and sensitivity in detecting lesions, but it requires more prospective clinical trials (75). Recent research focused on a group with prostate cancer, which was suitable for ^68^Ga-PSMA, and data prompts its appliance in primary prostate cancer to generally evaluate tumor burden and metastases.

Aggressive prostate cancer, however, transforms into higher glucose metabolism and makes it possible for ^18^F-FDG PET/CT to detect tumors. In high-risk prostate cancer, ^18^F-FDG still has limitations on low sensitivity while ^18^F-FDG is competitive to ^68^Ga-PSMA in recurrence or CRPC. Both ^18^F-FDG and ^68^Ga-PSMA are predictors of prognosis and therapeutic effect. ^68^Ga-PSMA PET/CT is the mainstream of nuclear imaging in CRPC and mCRPC. The high sensitivity and specificity make it promising in treatment management. While partial patients are PSMA negative, which increases the difficulty in disease surveillance. Some characters may be found such as low serum PSA level, special pathological subtype, or different molecular mechanisms. Several clinical trials revealed ^18^F-FDG could effectively detect metastases after ADT. Tumors in CRPC and mCRPC generally elevate glucose metabolism, and FDG can localize the recurrence and metastases, which are negative in PSMA. Although ^68^Ga-PSMA is effective in evaluating recurrence, and ^18^F-FDG PET/CT can compensate for the shortage and effectively verify the tumor lesions in CRPC.

## Data Availability Statement

The original contributions presented in the study are included in the article/supplementary material, further inquiries can be directed to the corresponding authors.

## Ethics Statement

Written informed consent was obtained from the individual(s) for the publication of any potentially identifiable images or data included in this article.

## Author Contributions

KS and BL wrote the first manuscript. KS and BL contributed equally as first authors. YJ, LC, and XZ read, reviewed, edited, and wrote sections related to their areas of expertise. WX and QW read, reviewed, edited throughout the whole writing process, and signed off on the final paper. All authors contributed to the article and approved the submitted version.

## Funding

We are grateful for funding provided by the National Natural Science Foundation of China (No 81702542, 81972578, 82072847, and 81772742); Science and Technology Commission of Shanghai Municipality (19XD1402300); Shanghai Municipal Health Commission (2019LJ11); Shanghai Jiao Tong University Medical Engineering Cross Fund (YG2019GD02).

## Conflict of Interest

The authors declare that the research was conducted in the absence of any commercial or financial relationships that could be construed as a potential conflict of interest.

## Publisher’s Note

All claims expressed in this article are solely those of the authors and do not necessarily represent those of their affiliated organizations, or those of the publisher, the editors and the reviewers. Any product that may be evaluated in this article, or claim that may be made by its manufacturer, is not guaranteed or endorsed by the publisher.
